# Radiographic Investigation of Root Canal Morphology of Permanent Mandibular Molars in Makkah Population (Saudi Arabia) Using Cone-Beam Computed Tomography

**DOI:** 10.1155/2022/1535752

**Published:** 2022-06-03

**Authors:** Laila Mohamed Mohamed Kenawi, Raghad Fahad Althobaiti, Dina Mohammad Filimban, Sarra Dakheel Allah Alotaiby, Majedh Awad Alharbi, Wed Mohammed Kassar

**Affiliations:** ^1^Department of Endodontics, Faculty of Dentistry, Cairo University, Cairo, Egypt; ^2^Department of Conservative and Restorative Dentistry, Faculty of Dentistry, Umm Al Qura University, Makkah, Saudi Arabia; ^3^South of Al-Madinah Primary Health Care Center, Besha 67713, Saudi Arabia; ^4^Hubona General Hospital, Hubona 6632, Saudi Arabia; ^5^Al Safwa Dental Clinic, Makkah 24414, Saudi Arabia; ^6^Bani Farwa Primary Health Care Center, Al-Baha, Saudi Arabia

## Abstract

**Objectives:**

This study aimed to analyze the root canal morphology of first and second permanent mandibular molars among Makkah population by using cone-beam computed tomography (CBCT).

**Materials and Methods:**

CBCT images of two hundred eight patients were obtained from the Faculty of Dentistry, Umm Al-Qura University, Makkah, Saudi Arabia. In all images, mandibular first and second molars were evaluated for the number of roots and their morphology, number of root canals, canal configuration in each root according to Vertucci's classification, and the presence of a C-shaped canal.

**Results:**

Most of the mandibular first molars had two roots (96.5%) and three root canals (77.7%). Type IV canal configuration prevailed (75.6%) in the mesial roots, and type I canal configuration prevailed (71.7%) in the distal roots. For the mandibular second molars, the majority had two roots (96.3%) and three root canals (82.7%). Type IV canal configuration prevailed (48%) in the mesial roots and type I (99.1%) in the distal roots. C-shaped canals were detected in 5.7% of the mandibular first molars and 4% of the mandibular second molars. There was no statistical difference between the mandibular first and second molars (*P* > 0.05) in the shape of the roots and the presence of the C-shaped canals.

**Conclusion:**

Most of the mandibular molars in Makkah population presented with two roots and three root canals. The incidence of three-rooted mandibular molars was low, and it was higher in the first molars than second molars. The type IV root canal system prevailed in mesial roots and type I in distal roots.

## 1. Introduction

The knowledge of the root canal morphology is necessary for successful root canal treatment. It is important to be familiar with variations in tooth anatomy and characteristic features in various racial groups as this knowledge can aid in location, negotiation, and management of canals during root canal treatment [1]. Due to the complexity of the root canal system, more than one classification was suggested to describe it. One of these classifications is the one described by Vertucci [2].

The mandibular molars usually have two roots with three or more root canals: two mesial and one or two distal root canals [3, 4]. Studies showed morphological variations in the mandibular molars as the occurrence of a third mesial canal, called the middle mesial canal [2, 5]. There is a racial variation in the frequency of C-shaped molars which ranged between 10 and 32.7% [6, 7]. Several methods were used for the detection of root canal anatomy, including the tooth-clearing technique, root sectioning, micro-computed tomography (micro-CT), cone-beam computed tomography (CBCT), radiographic examination, and magnifying loupes [1, 2, 8–11]. Magnetic resonance imaging (MRI) was also suggested as a possible non-radiographic imaging technique for the visualization of endodontic anatomy [12]. The morphology of the mandibular first molars was assessed in more than one Saudi population. In Al-Medina Al-Munawarah population, the root canal morphology and its variation in mandibular first molar teeth were assessed using CBCT. The results showed that most of the teeth had two separated roots with a high percentage of two canals in the mesial root and a distal root with one canal. Also, the frequency of C-shaped canals was very low [9].

One study investigated the number of root canals in endodontically treated mandibular first molars clinically and radiographically at the University of King Saud College of Dentistry in Riyadh. The results showed that 57.76% of the evaluated teeth had four root canals (two mesial and two distal) and 42.3% had three root canals (two mesial and one distal). The authors concluded that the incidence of four root canals in the mandibular first molar of a Saudi subpopulation was high [10].

The variation in root canal morphology of mandibular first molars in the Saudi Asir region was assessed by using two periapical X-rays in different angulations for each patient seeking root canal treatment, then access cavity preparation was performed, and magnifying loupes were used to locate the canals. The author found that 70% of cases had three canals, 29% had four canals, and only 1% presented with extra-distolingual root [11].

In Al-Jouf region of Saudi Arabia, a study evaluated the incidence of three roots and four canals in mandibular first permanent molars. They found that only six teeth from 100 had three roots and 63% had four root canals [13]. Another study found that the incidence of three-rooted mandibular first molars in this population was only 3.27% by CBCT. The authors concluded that a third root can be present in mandibular first molars and that CBCT can aid in the accurate diagnosis of the presence of a third root [14].

Few studies are available on the morphology of mandibular molars in the Saudi population using CBCT, especially in Makkah region. Therefore, the aim of the present study was to analyze the root canal morphology of first and second permanent mandibular molars among Makkah population by using cone-beam computed tomography (CBCT).

## 2. Materials and Methods

This study was conducted to evaluate the root canal morphology of first and second permanent mandibular molars using CBCT images, performed at the Faculty of Dentistry, Umm Al-Qura University, Makkah, Saudi Arabia, for different dental purposes in the period from 2013 to 2018.

The CBCT images were obtained by an experienced radiologist using a CBCT scanner (I-CAT Vision TM; Imaging Science International, Hatfield, PA, USA) at 120 kVp and 37.07 mA with an exposure time of 26.9 s. The voxel size of the images was 0.25 mm.

### 2.1. Selection of the Sample

CBCT images of 208 Saudi patients of both sexes, aged between 18 and 55 years, were included in the study. For each patient, age and gender were recorded.

In all images, right and left first and second permanent mandibular molars were evaluated. Inclusion criteria were clear, undistorted CBCT images, fully erupted first and second permanent mandibular molars, with completely formed roots. In contrast, retained deciduous molars, teeth with open apices, or root resorption as well as endodontically treated molars were excluded from the study.

### 2.2. Method

**Figure 1:**
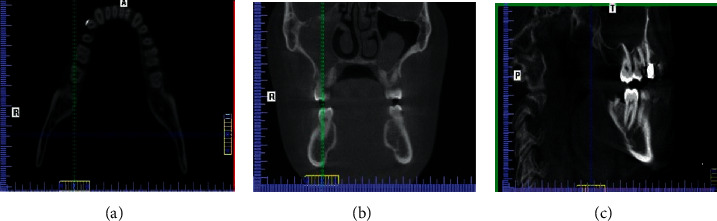
Cone-beam computed tomography images. (a) Axial view. (b) Coronal view. (c) Sagittal view.

**Figure 2:**
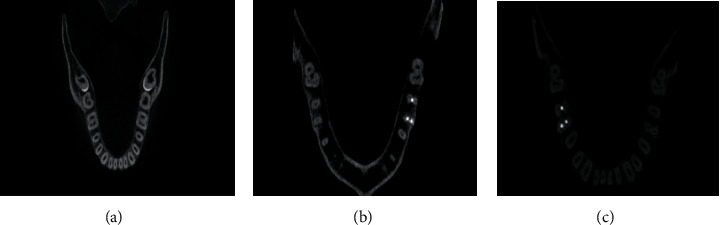
Axial CBCT sections for C-shaped canals. (a) Type C1 in tooth #47. (b) Type C2 in tooth #37. (c) Type C2 in tooth #47.

Each CBCT image was observed separately by two examiners, and any disagreement between them was discussed until a consensus was reached. The roots of right and left first and second permanent mandibular molars were examined in three views: sagittal, coronal, and axial (Figure 1), and in the axial view, roots were examined at the cervical, middle, and apical levels. For each molar, the number of roots and their morphology, number of canals per root, canal configuration in each root, and the presence of the C-shaped canal were recorded (Figure 2). Vertucci's classification for root canal configurations was used in this study [2]. And, C-shaped canals of mandibular molars were classified according to Fan criteria [15].

### 2.3. Statistical Analysis

Data were tabulated and statistically analyzed using IBM SPSS Statistics v. 22. The chi-square test was used for analysis with a *P* value of 0.05. A multivariable comparison was made using customized tables, and single variables were analyzed by cross-tabulation.

## 3. Results

CBCT images of 630 mandibular molars (283 mandibular first molars and 347 mandibular second molars) from 208 Saudi patients were evaluated in this study.

### 3.1. Number of Roots and Their Morphology

#### 3.1.1. The Number of Roots

**Figure 3:**
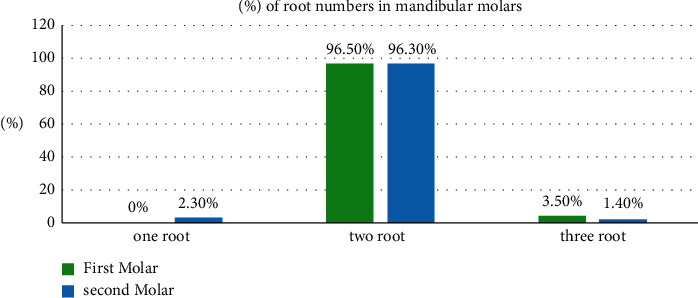
A bar chart showing the frequency of the numbers of roots in the mandibular molars.

None of the evaluated mandibular first molars had a single root, 96.5% had two roots, and only 3.5% had three roots (Table 1, Figure 3).

Mandibular second molars evaluation revealed that (2.3%) had one root, 96.3% had two roots, and only 1.4% had three roots (Table 1, Figure 3).

Using the chi-square test, there was a statistically significant difference between the mandibular first and second molars (*P* < 0.05) in the number of roots (Table 1).

#### 3.1.2. Root Morphology

As for the shape of the roots in the mandibular first molar, 13.4% of the mesial roots were curved and 1.4% were dilacerated. 2.5% of the distal roots were curved, and only 0.4% were dilacerated. Neither of the mesial nor the distal roots had an S-shaped curve.

Regarding the mandibular second molars, 13.6% of the mesial roots were curved, 2.4% were dilacerated, and 1.5% were S-shaped. As for the distal roots, 3.2% were curved, 0.3% had dilacerations, and 0.3% were S-shaped.

There was no statistical difference between the mandibular first and second molars (*P* > 0.05) in the shape of the roots as shown in Table 2.

### 3.2. The Number of Root Canals

**Figure 4:**
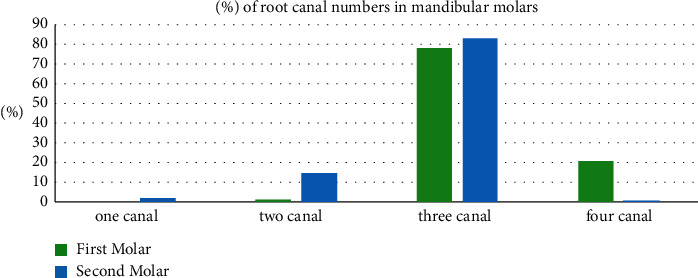
A bar chart showing the percentage of the number of root canals in the mandibular molars.

The mandibular first molar evaluation revealed that none of the mandibular first molars had one canal, 1.4% had two canals, 77.7% had three canals, and 20.8% had four canals (Table 3, Figure 4).

Regarding the root canal distribution, about 97.5% of the examined mandibular first molars had two mesial canals (mesiobuccal and mesiolingual). A single distal canal was found in more than 71% of the examined first molars.

The mandibular second molar evaluation revealed that 2% had one canal, 14.7% had two canals, 82.7% had three canals, and 0.6% had four canals (Table 3, Figure 4).

Regarding the root canal distribution in mandibular second molars having more than one root, nearly 85% had two mesial canals (mesiobuccal and mesiolingual) and 99% had a single distal canal.

To study the variation between the mandibular first and second molars in the number of canals, the chi-square test was used (Table 3 and Figure 4).

There was a statistically significant difference between the mandibular first and second molars (*P* < 0.05) in the number of canals.

### 3.3. The Canal Configuration in Each Root

The most prevalent canal configuration for the mandibular first molar mesial roots was type IV (75.6%), followed by type II (20.1%), type I (2.1%), type III (1.4%), and finally type V and type VI (0.4%). However, type I prevailed in distal roots (71.7%), followed by type III (11.0%), type IV (7.8%), type II (6.7%), and type V (2.8%).

For mandibular second molars having more than one root, type IV canal configuration was the most frequent in the mesial roots with 48% followed by type II (31.3%), type I (14.2%), type III (4.7%), and type V (1.8%), while type I prevailed in distal roots with 99.1%.

**Figure 5:**
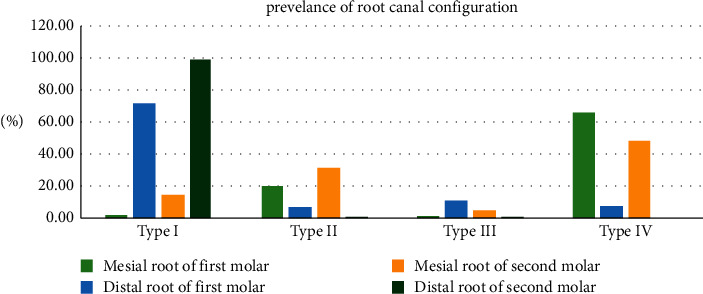
A bar chart showing the percentage of each type of root canal configurations in mesial and distal roots of mandibular first and second molars.

Mesial and distal canal configurations statistically differ between the first and the second molars (*P* < 0.05) (Table 4, Figure 5).

For mandibular second molars, type I canal configuration was the most prevalent when a single root was presented (50%), followed by type III (25%), type II, and type V (12.5%).

### 3.4. Presence of the C-shaped Canal

The results indicated that 5.7% of mandibular first molars had C-shaped canals. 87.5% of the C-shaped canals were C1, and 12.5% were C2, while 4% of the mandibular second molar had C-shaped canals of which 92.9% were C1, and 7.1% were C2. There was no statistical difference between the mandibular first and second molars (*P* > 0.05) in the presence of C-shaped canals as shown in Table 5.

## 4. Discussion

An essential factor in the success of endodontic treatment is the understanding of the root and canal morphology, as the complexity of the root canal system determines the difficulty of root canal treatment [13]. If a root canal is missed, the infected pulp tissue and microorganisms left will result in failure [16]. Many methods were used to assess root canal morphology. In this study, we used cone-beam computed tomography, as it is a non-invasive, accurate diagnostic imaging modality and it has the ability to provide a three-dimensional image of the teeth and related anatomical structures [14]. It can identify a greater number of root canal systems than digital periapical radiography [17]. The classification of Vertucci was taken as a reference to describe the root canal system as it is the most commonly used classification [18, 19].

This study provides detailed information regarding the root and canal morphology of permanent mandibular first and second molars in Makkah population in Saudi Arabia. Mandibular first permanent molars are generally described as two-rooted teeth with three canals [2, 9]. In this study, the majority (96.5%) of the examined mandibular first molars had two roots; our findings coincide with the results of other studies on other Saudi populations [9, 10, 19, 20], as well as Egyptian [18], South African [21], Turkish [22, 23], Indian [24], Jordanian [25], Yemeni [26], Belgian and Chilean [27] populations, where 2-rooted mandibular first molars dominated. Only 3.5% had three roots. This was almost similar to the previously reported prevalence in other Saudi [14, 20, 28], Sudanese [1], and Iranian [29] populations and slightly lower than the percentages (5.97% [10] and 5.5% [19, 30]) reported in other regions of Saudi Arabia and far less than the percentages reported by De Pablo et al. [5] (13%) in their systematic review, as well as in Thai (12.7%) [31], Taiwanese (33.33%) [32], and Chinese (29%) [33] populations. However, our findings were slightly higher than those reported in South African population (1%) [21].

Regarding the permanent mandibular second molars, two-rooted molars prevailed in 96.3%. The frequency of two separate roots was close to that found in an Egyptian population (98.16%) [18] but higher than the incidence reported in another Saudi population (89.6%) [19] and in Thai (54%) [31], Sudanese (78%) [1], Chinese (76%) [33], Burmese (58.2%) [34], Turkish (85.4%) [23], and Iranian (88.8%) [35] populations. In our study, only 1.4% of the permanent mandibular second molars had three roots; this was similar to other Saudi subpopulations (1.4% [28] and 1.7% [19]) and lower than those of Sudanese (3%) [1], Turkish (3.45%) [23], and Iranian (9.2%) [35] populations. 2.3% of the examined mandibular second molars had one root, which coincided with the reported incidence for Egyptian (2.86%) [18], Jordanian (2%) [25], and Iranian (2%) [35] populations and was slightly higher than the Turkish population (1.29%) [23]. However, it was lower than the reported incidence for Saudi (8.5%) [19], Thai (10%) [31], and Chinese (22%) [33] populations.

Our results revealed that the incidence of three-rooted mandibular molars was higher in the first molars than that in the second molars, and this was consistent with the findings by Riyahi et al. in 2019 [28] and Mashyakhy et al. in 2021 [19] in other areas of Saudi Arabia, and with Zhang et al. 2011 [33] in China, Torres et al. 2015 [27] in Belgian and Chilean populations, and Ngeow et al. 2020 [36] in the Malays. Moreover, the incidence of single-rooted molars was higher in the second molars than that in the first molars as found by other studies [18, 19, 23, 33].

In the present study, the majority (77.7%) of mandibular first molars had three canals, like in other Saudi populations where three-canaled mandibular first molars dominated but with lower percentages (73% [20], 70% [11], and 64.5% [19]). This was also in agreement with the findings of studies conducted on Egyptian [18], Thai [31], Turkish [23], and Brazilian [37] populations where three-canaled first molars were the most common ones. However, our findings were different from those of Al-Nazhan who reported that the incidence of mandibular first molars with four root canals in a Saudi subpopulation was 57.76% and found it higher than that of three root canals (42.3%) [10]. Regarding the canal distribution, the mandibular first molar had a higher occurrence of 2 canals in the mesial root and one canal in the distal root, and this was in accordance with other studies on different populations [9, 22, 24, 26, 37, 38].

In the mandibular second molars, the presence of three canals dominated with an incidence of 82.7%, which was in accordance with the results of a study conducted on another Saudi population (80.4%) [19], but it was higher than the incidence found in Turkish (72.8%) [23], Jordanian (58%) [25], Thai (58%) [31], and Brazilian (54%) [37] populations. Regarding the canal distribution, we found that most of the mandibular second molars had 2 mesial canals and a single distal canal, and this was in accordance with the findings reported for different populations [1, 23, 25, 37].

In the current study, the most common canal configuration, for mandibular first molar mesial roots, was type IV (75.6%), followed by type II (20.1%). These findings agreed with the results of de Pablo et al. in their systematic review [5], and studies in other Saudi populations [19, 20], as well as studies in Sudanese [1], Egyptian [18], South African [21], Turkish [22], and Jordanian [25] populations, they found type IV to be the most prevalent canal configuration in the mesial root followed by type II. However, our findings were different from those of Zafar and Alrahabi 2015 [9] in Al-Medina Al-Munawarah in Saudi Arabia and Senan et al. 2020 [26] in Yemen; they reported that the most common canal configuration for the mesial roots was type II, and John et al. 2021 [39] in India reported that more than two thirds of the subjects had type VI mesial root canal configuration. Regarding the distal roots of mandibular first molars, type I was the most prevalent (71.7%). These findings were in accordance with the studies conducted in different areas of the world where type I dominated in distal roots with variable percentages [1, 5, 9, 19–21, 24, 27, 38, and 39] but were in disagreement with the findings by Senan et al. in Yemen [26], as they found that type III was more common in distal roots of mandibular first molars.

For mandibular second molars having more than one root, type IV canal configuration prevailed in the mesial roots with 48.1%, followed by type II (31.3%), type I (14.2%), type III (4.7%), and type V (1.8%). Our findings were in agreement with the findings of Ahmed et al. in Sudan [1], Mashyakhy et al. in Saudi Arabia [19], Al-Qudah and Awawdeh in Jordan [25], and Gulabivala et al. in Thailand [31]; they all reported that the most common canal configurations of mesial roots of mandibular second molars were type IV followed by type II, similar to findings of Demirbuga et al. in Turkey [23] who found type IV to dominate in the mesial roots. However, our findings were different from other studies conducted in Egypt [18], Iran [35], and Iraq [40] where type II was more common in mesial roots of mandibular second molars. Type I canal configuration prevailed (99.1%) in the distal roots of mandibular second molars. This was in accordance with the results of studies, from different regions of the world, which reported that type I was the most common canal configuration in the mandibular second molar distal root [1, 18, 19, 23, 25, 27, 31, 33, 35, and 40].

For single-rooted mandibular second molars, type I canal configuration was the most prevalent (50%), followed by type III (25%). This was in contrast with the findings of Al-Qudah and Awawdeh who reported teeth with a single conical root to have 100% type I root canal configuration [25].

In the present study, the incidence of C-shaped root canal configurations in mandibular first molars in Makkah population was 5.7%; this was higher than the results reported in other Saudi populations by Alfawaz et al. (0.19%) [41] and Mashyakhy et al. [42] who found that C-shaped canals were absent in the first molars; it was also higher than the percentages reported in other parts of the world like Brazilian (1.7%) [37], Portuguese (0.6%) [43], and Indian (0.2%) [44] populations. It was close to the incidence reported in the Malays (4.3%) [36]. However, it was lower than the incidence (24.01%) reported in another study in Brazil [45].

The incidence of mandibular second molars with C-shaped root canals was reported from 2.7 to 44.5% [46]. Asian populations showed the highest incidence, specifically in Korean (44.5%) [47]and Chinese (29–39%) populations [48]. However, the prevalence in Europe was 10% in Belgium [27] and 8.5% in Portugal [43]. In Brazil, the prevalence was 21.32% [45] while it was 8.2% in the Indian [44] population. In the Middle East, a prevalence of 10% was reported in several studies, as in Sudanese [1] and Jordanian [25] populations, and it ranged from 7.9% to 25.5% in Saudi populations [6, 41, 42, 49]and was reported to be 9.2% in the Iranian population [35]. In our study, the prevalence was found to be 4%, which is lower than the prevalence found in many parts of the world.

The type of C-shaped root canal anatomy in Saudi Arabia was evaluated using CBCT by several studies; they found that the C3 canal configuration was the most common type [6, 41, 42]. This was in contrast to the finding of the current study where C1 canal configuration was the most common type.

Regarding the shape of the roots in the present study, the prevalence of the root curvature was found to be higher in the mesial roots (13.4%) than that found in the distal roots of mandibular first molars (2.5%). As for the second molars, the prevalence of the curvature in the mesial roots (13.6%) was higher than it was for the distal roots (3.2%). These findings were in accordance with the study by Ahmed et al. [1] who showed a higher prevalence of the root curvature of the first molar in the mesial roots than that in the distal roots, but with different percentages, in a Sudanese population and found that the second molar had 3% of the distal roots curved distally.

The prevelance of dilacerated roots in the first and second mandibular molars was low, similar to the results from different studies including Croatian and Turkish populations [50, 51].

## 5. Conclusions

In the present study, the majority of mandibular molars in Makkah population had two roots. The incidence of three-rooted mandibular molars was low, and it was higher in the first molars than second molars.

Regarding the root canal number and their distribution, most mandibular molars had three canals, with two canals in the mesial root and one canal in the distal root.

Type IV and type I canal configurations were the most prevalent in the mesial and distal roots, respectively, of both mandibular first and second molar teeth.

The C-shaped canal configuration was commonly seen in both first and second mandibular molars, in which type C1 prevailed.

## Figures and Tables

**Table 1:** Frequency of the numbers of roots in permanent mandibular molars.

	First molar (%)	Second molar (%)	*P*value
Number of roots			
One root	0	2.3	0.009
Two roots	96.5	96.3	
Three roots	3.5	1.4	

**Table 2:** The frequency distribution and percentage of the root shapes in the first and second permanent mandibular molars.

Root morphology	First molar mesial root (%)	Second molar mesial root (%)	First molar distal root (%)	Second molar distal root (%)
Curved	13.4	13.6	2.5	3.2
Dilacerated	1.4	2.4	0.4	0.3
S-shaped	0	1.5	0	0.3
*P*value	0.074		0.667	

**Table 3:** Frequency of the numbers of root canals in mandibular molars.

	First molar (%)	Second molar (%)	*P*value
Number of canals			
One canal	0	2.0	≤0.001
Two canals	1.4	14.7	
Three canals	77.7	82.7	
Four canals	20.8	0.6	

**Table 4:** Frequency and distribution of root canal configurations according to Vertucci's classification.

Canal configurations	The mesial root of mandibular first molar (%)	The mesial root of the mandibular second molar (%)	The distal root of the mandibular first molar (%)	The distal root of the mandibular second molar (%)
Type I	2.1	14.2	71.7	99.1
Type II	20.1	31.3	6.7	0.3
Type III	1.4	4.7	11.0	0.3
Type IV	75.6	48	7.8	0.0
Type V	0.4	1.8	2.8	0.3
Type VI	0.4	0.0	0.0	0.0
*P*value	≤0.001		≤0.001	

**Table 5:** The frequency of C-shaped canals.

Root shape	First molar (%)	Second molar (%)	Chi-2	*P* value
C1	87.5	92.9	1.01	0.604
C2	12.5	7.1		
Total number of C-shaped frequency	5.7	4	0.901	0.354

## Data Availability

The data are available upon request from the corresponding author.
